# Ingestion of huge number of metallic nails impacted in the stomach and cecum in a mentally abnormal woman: Case report

**DOI:** 10.1016/j.ijscr.2020.04.019

**Published:** 2020-05-07

**Authors:** Ayad Ahmad Mohammed

**Affiliations:** Department of Surgery, College of Medicine, University of Duhok, Azadi Teaching Hospital, 8 Nakhoshkhana Road, 1014 AM, Duhok, Kurdistan Region, Iraq

**Keywords:** Foreign bodies, Metallic nails, Laparotomy, Acute abdomen, Endoscopy, Plain abdominal X-ray

## Abstract

**Background:**

Foreign bodies may be ingested unconsciously, intentionally or in patients who have psychological abnormalities. The majority of foreign bodies are passed smoothly with no problems if they passed the esophagus, however some may be lodged in areas of anatomical narrowing which may require endoscopic or surgical intervention.

**Case presentation:**

A 37-year -old mentally abnormal woman presented to the emergency department complaining from abdominal pain for 2 days. The family reported a history of ingestion of multiple metallic nails one week before presentation. Abdominal examination showed generalized tenderness and guarding. Plain abdominal X-ray showed multiple metallic nails in the upper abdomen and the right lower abdomen. Laparotomy was performed, one of the nails was causing perforation of the gastric wall, huge number of metallic nails were extracted from the stomach and cecum, appendectomy was performed.

**Conclusion:**

Ingestion of sharp and long foreign bodies usually requires surgical intervention, after surgery close observation and psychological consultation is required to prevent repeated ingestions.

## Highlights

• Foreign bodies may be ingested unconsciously, intentionally or in patients who have psychological abnormalities.
• The majority of foreign bodies are passed smoothly with no problems.
• Ingestion of sharp and long foreign bodies usually requires surgical intervention.

## 1 Introduction

Foreign body ingestion is a potentially serious health problem. The majority of the affected individuals are among the pediatric age groups. In adults, foreign bodies may be ingested unconsciously, intentionally or in patients who have psychological abnormalities or alcohol dependence [[1], [2], [3]].

The exact incidence of foreign bodies ingestion is not very well reported due to the wide variety of its types, patients may ingest bizarre objects like sharp instruments, nails, magnets, hair, or any other object [4,5].

The majority of foreign bodies are passed smoothly with no problems if they passed the esophagus, however some may be lodged in areas of anatomical narrowing such as the crico-pharyngeus, the lower esophageal sphincter, the pyloric canal, the ileo-caecal region and the anus. About 10–29 % may require endoscopic intervention. Surprisingly surgical intervention is required in the minority of them [3,6,7].

Perforations of the stomach, and bowel may be caused by long foreign bodies, blunted foreign bodies may also cause perforation by pressure necrosis. The most common sites for perforation are the esophagus and the terminal ileum [1,3,6].

Metallic foreign bodies when ingested are usually evident on plain abdominal X-rays. Other modalities of radiology may be required when complications are suspected such as perforation [8].

The treatment depends on the mode of presentation, the nature of foreign body, and the anatomical site of impaction.

This report is in line with the SCARE 2018 criteria [9].

## 2 Patient information

A 37-year -old mentally abnormal woman presented to the emergency department complaining from abdominal pain for 2 days. The pain was mainly in the upper abdomen, associated with nausea, no vomiting. The family reported a history of ingestion of multiple metallic nails one week before presentation.

The patient was a known case mental abnormality and was on treatment for the last 15 years, the past surgical history was negative.

### 2.1 Clinical findings

During examination the pulse rate was 110 beats per minute, the blood pressure was 110/60 mmHg, and the temperature was 38.2 degrees of Celsius.

Abdominal examination: showed generalized tenderness and guarding. There were no jaundice or pallor.

### 2.2 Diagnostic assessment

**Fig. 1:**
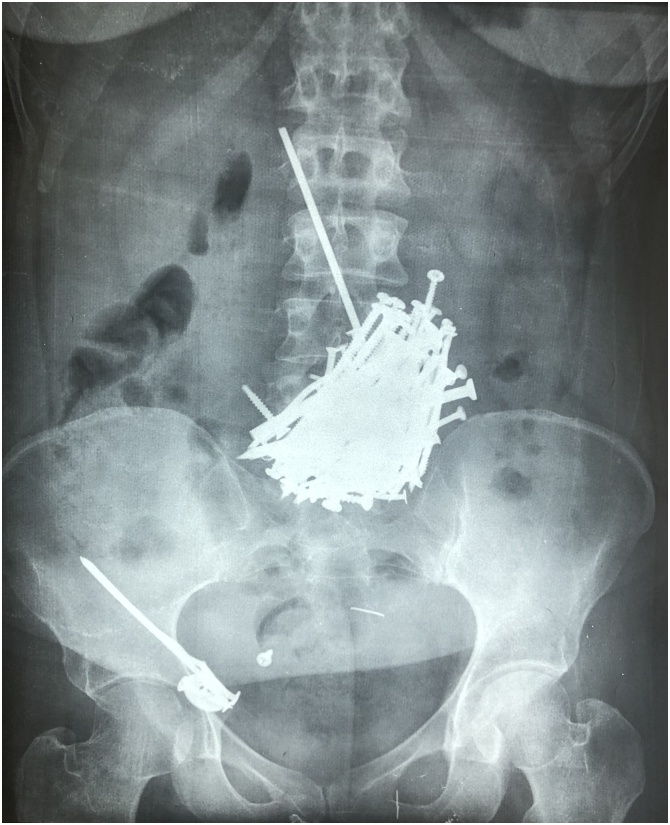
Plain abdominal X-rays of the abdomen showing multiple metallic nails in the stomach and the terminal ileum.

Plain abdominal X-ray showed multiple metallic nails in the upper abdomen and the right lower abdomen Fig. 1.

### 2.3 Therapeutic intervention

**Fig. 2:**
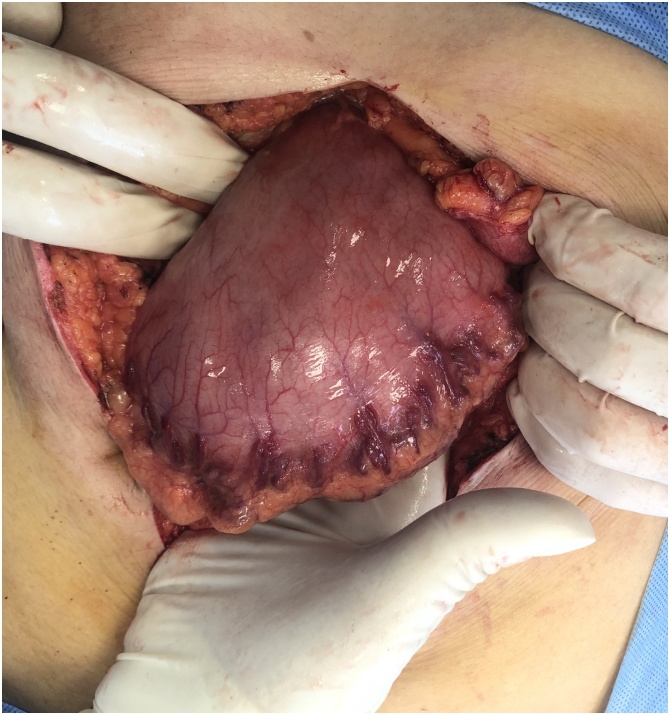
An intraoperative picture showing the stomach filled with large number of metallic nails.

**Fig. 3:**
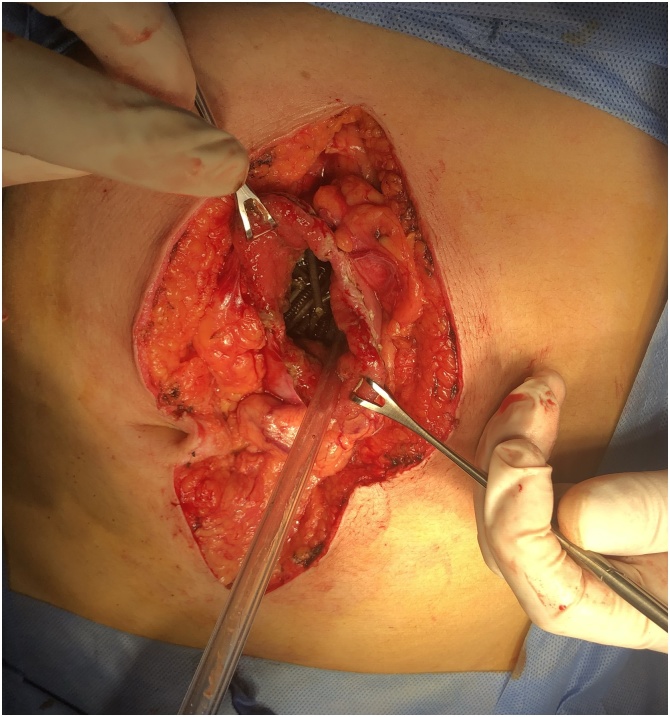
An intraoperative picture showing the gastric cavity with containing the metallic nails.

**Fig. 4:**
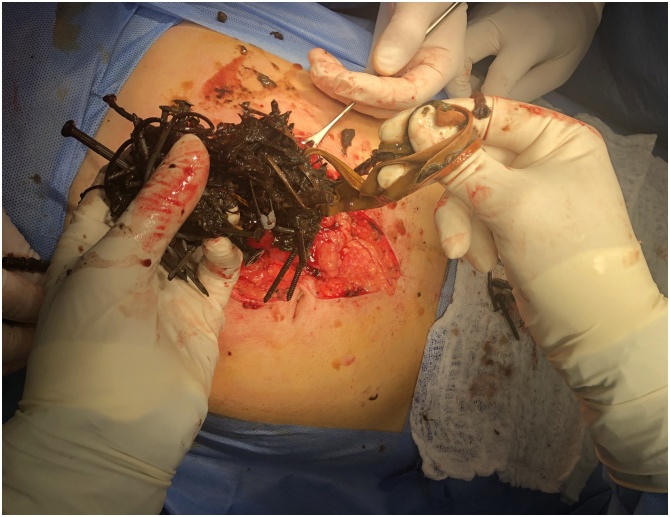
An intraoperative picture showing the extraction of the nails from the stomach.

**Fig. 5:**
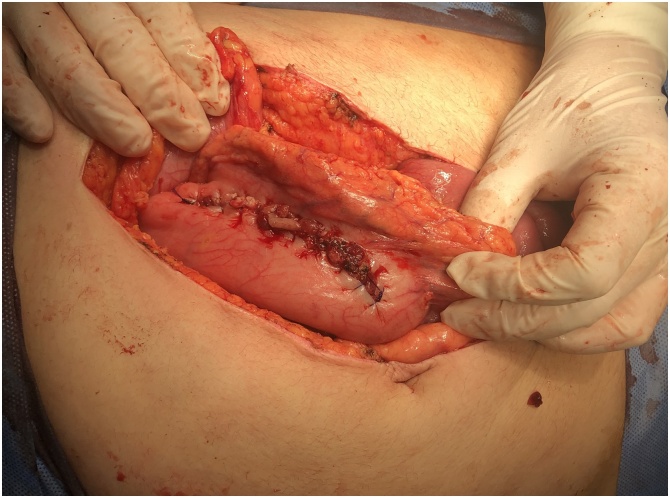
An intraoperative picture showing the closure of the stomach using a slowly absorbable suture material.

Resuscitation was done and the patient referred to the operation room for laparotomy. Laparotomy was performed with a midline incision, one of the nails was causing perforation of the gastric wall. The stomach was opened with longitudinal incision, huge number of metallic nails were extracted from the stomach, the stomach closed in 2 layers with a slowly absorbable suture material Fig. 2, Fig. 3, Fig. 4, Fig. 5.

**Fig. 6:**
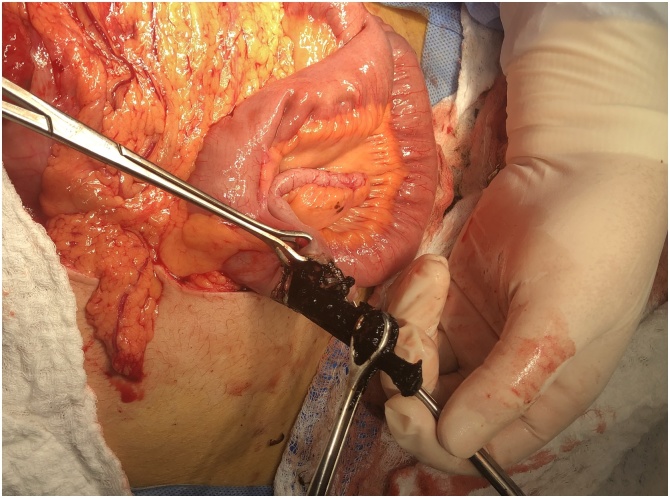
An intraoperative picture showing appendicostomy and the extraction of metallic nails from the cecum.

**Fig. 7:**
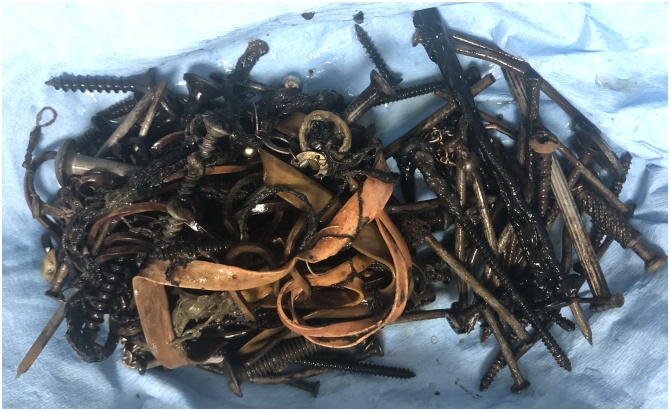
A picture showing the metallic nail, some elastic rubbers, and safety pins which were extracted from the gastric and caecal lumens.

The cecum was opened near the base of the appendix, extraction of multiple nails and appendectomy were performed. The abdomen was irrigated with warm saline and tube drain in the abdominal cavity, gastric tube was inserted during surgery Figs. 6 & 7 .

### 2.4 Follow-up and outcomes

The patient was admitted for 5 days after surgery with smooth postoperative period, the nasogastric tube was removed after 2 days and oral intake was started at the 3rd day. The patient was in-patient for 5 days and patient was discharged on the 5th day with no complications.

## 3 Discussion

Ingested metallic objects is a common medical problem that is sometimes seen in the emergency departments, metallic coins are among the most commonly ingested foreign bodies [3,8].

Impaction usually occur in areas of anatomical narrowing, they may be impacted also in anatomical sites where curvatures are present like the duodenal curvature, or sites of pathological strictures like inflammatory, or after surgeries from adhesions [3].

Generally, large foreign bodies will not pass the pyloric canal and similarly long greater ones may not progress beyond the duodenal curvature [3].

Some metallic forging bodies can be extracted using endoscopy especially if they are small and in the stomach using forceps. Magnets can be also used to extract the metallic foreign bodies when passed the duodenum. When they passed the duodenum they may be followed by X-rays and may passed with stool [10].

Some authors showed that the ability of hand held metal detectors for the identification of ingested foreign bodies may reach 90 %, these devices are useful when they are present in many segments of the bowel [11].

When foreign bodies pass the pylorus, they may be followed by weekly radiographs provided that the patient is clinically stable and has no abdominal pain. The majority will pass within one week if not causing perforation. When repeated X-rays show no progress of the foreign body they may require intervention [3].

Management of metallic foreign bodies in the terminal ileum are usually extracted using appendicostomy is the safest method [2].

Batteries, whether disc or button batteries, usually require urgent endoscopic intervention because of the risk of chemical burn, liquefaction necrosis and subsequent perforation [3].

In this case the indication for surgery was failure of progress and impaction in the stomach, the patient has acute abdomen at presentation suggesting perforation.

## 4 Conclusion

Ingestion of sharp and long foreign bodies usually requires surgical intervention, after surgery close observation and psychological consultation is required to prevent repeated ingestions.

## Declaration of Competing Interest

The author has no conflicts of interest to declare.

## Sources of funding

None.

## Ethical approval

Ethical approval has been exempted by my institution for reporting this case.

## Consent

An informed written consent was taken from the family for reporting the case and the accompanying images because the patient is mentally not competent.

## Author contribution

The concept of reporting the case, data recording, and drafting the work done by Dr Ayad Ahmad Mohammed.

Dr Ayad Ahmad Mohammed took the consent from the patient for publishing the case.

Final approval of the work to be published was done by Dr Ayad Ahmad Mohammed.

## Registration of research studies

This work is case report and there is no need of registration.

## Guarantor

Dr Ayad Ahmad Mohammed is guarantor for the work.

## Provenance and peer review

Not commissioned, externally peer-reviewed.
